# Fault Diagnosis Method for a Mine Hoist in the Internet of Things Environment

**DOI:** 10.3390/s18061920

**Published:** 2018-06-13

**Authors:** Juanli Li, Jiacheng Xie, Zhaojian Yang, Junjie Li

**Affiliations:** 1 Shanxi Key Laboratory of Fully Mechanized Coal Mining Equipment, College of Mechanical Engineering, Taiyuan University of Technology, Taiyuan 030024, China; xiejiacheng0007@link.tyut.edu.cn (J.X.); yangzhaojian@tyut.edu.cn (Z.Y.); lijunjie0051@link.tyut.edu.cn (J.L.); 2 Post-Doctoral Scientific Research Station, Shanxi Coking Coal Group Co., Ltd., Taiyuan 030024, China

**Keywords:** Internet of Things (IoT), mine hoist, fault diagnosis, ZigBee, Dezert-Smarandache Theory (DSmT)

## Abstract

To reduce the difficulty of acquiring and transmitting data in mining hoist fault diagnosis systems and to mitigate the low efficiency and unreasonable reasoning process problems, a fault diagnosis method for mine hoisting equipment based on the Internet of Things (IoT) is proposed in this study. The IoT requires three basic architectural layers: a perception layer, network layer, and application layer. In the perception layer, we designed a collaborative acquisition system based on the ZigBee short distance wireless communication technology for key components of the mine hoisting equipment. Real-time data acquisition was achieved, and a network layer was created by using long-distance wireless General Packet Radio Service (GPRS) transmission. The transmission and reception platforms for remote data transmission were able to transmit data in real time. A fault diagnosis reasoning method is proposed based on the improved Dezert-Smarandache Theory (DSmT) evidence theory, and fault diagnosis reasoning is performed. Based on interactive technology, a humanized and visualized fault diagnosis platform is created in the application layer. The method is then verified. A fault diagnosis test of the mine hoisting mechanism shows that the proposed diagnosis method obtains complete diagnostic data, and the diagnosis results have high accuracy and reliability.

## 1. Introduction

Mine hoisting work and the related operation status directly influence the safety of coal production and that of the operating personnel [1,2]. Studies on fault diagnosis methods for mine hoists are crucial to ensure safe, stable, reliable, and healthy mine operation. The application of artificial intelligence and other new technologies to fault diagnosis is being extensively studied. Vernekar used wavelet transform and support vector machine (SVM) to troubleshoot rolling bearings [3]. To determine the fault node set, a neural network was introduced into the fault diagnosis scheme, and this considerably improved diagnosis efficiency [4]. Li applied the class mean kernel principal component analysis algorithm to rolling bearing fault diagnosis to extract the class information of the data sample and accurately and quickly identify the fault mode [5]. Li studied an existing fault diagnosis system and the problems associated with intelligent fault diagnosis of the hoisting machine from the knowledge engineering viewpoint, such as the difficulties experienced with data acquisition, knowledge representation, and single fault diagnosis methods. This improved the efficiency and accuracy of reasoning [6]. Considering the challenges caused by coupling and the weak spindle system of the friction hoist, Dong et al. constructed a weighted undirected complex network model in which each data sample served as a node. This method accurately classified known fault types and recognized faults with high accuracy [7]. The data processing methods of these diagnostic studies are clear and effective in specific working environments, and they have been useful for mechanical fault diagnosis. However, these methods are mostly independent applications, and they do not use resources efficiently. The concept of the Internet of Things (IoT) diverges from the traditional thinking that has separated the information infrastructure from the physical infrastructure [8,9,10]. Under the framework of the three layers of the IoT—the perception layer, network layer, and application layer—a comprehensive intelligent and efficient diagnostic detection mode can be realized by using fault prediction, remote monitoring, remote diagnosis, and artificial intelligence.

The application of IoT technology to intelligent homes and agricultural intelligence is relatively mature, but the diagnosis of equipment failure based on IoT has not been researched widely. Feng et al. designed an intelligent wireless sensor network (WSN) connection mode with signal processing capability and verified its performance in bearing fault diagnosis to improve the transmission efficiency of WSN [11]. Based on the multi-level hierarchical information integration of WSNs, Tang proposed a mechanical fault diagnosis method so that a large number of vibration signals could be transmitted in real time during mechanical fault diagnosis [12]. Meanwhile, an artificial intelligence (AI) monitoring and diagnosis method based on WSN was preliminarily researched and applied [13,14,15,16,17]. Combining sensor technology, radio frequency technology, and intelligent processing technology with the industrial Ethernet, wireless sensor networks, and the Internet, a mine hoist perception system based on IoT technology was established [18,19]. This system was able to effectively monitor, manage, and remotely diagnose faults of the mine hoist system in real time. The reliability and fault diagnosis ability of the mine hoist system were also improved. Using IoT technology, a mine hoist monitoring system [20] was developed and applied to practical situations. The above research mainly applied IoT technology to remote monitoring.

For hoist fault diagnosis, the capabilities of the IoT in the field of remote monitoring have not yet been fully exploited, including features such as intelligence and interconnectedness. A complete IoT mine hoist fault diagnosis system has not yet been proposed. By fully exploiting the various characteristics of the IoT, such as information-awareness, network technology, and intelligent computing, real-time collaborative acquisition, intelligent processing, and timely feedback of mass information can be realized. In addition, a scheme for flexible, scalable, reconfigurable, and real-time interaction within the mine hoist fault mass information database can be established based on IoT. Based on these technologies, the dynamic adaptive fault feature component can be extracted to reflect the cause of the fault, thus improving the poor sensitivity of the common fault characteristics to mechanical faults, which has prevented effective fault diagnosis. By establishing a mine hoist fault diagnosis system based on the IoT, the causes of the failure of key mechanical parts of the mine hoist can be determined, allowing enterprises to quickly maintain and arrange production, and then fault information can be fed back to the manufacturing operation in a timely manner.

## 2. Architecture of the Mine Hoist Fault Diagnosis System Based on the IoT

**Figure 1:**
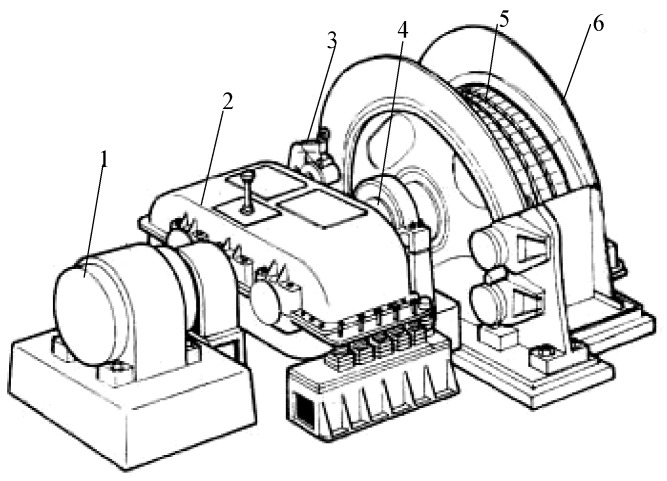
Multi-rope friction mining hoist: (1) motor, (2) reduction gearbox, (3) brake, (4) principal axis, (5) wire rope, and (6) guiding wheel.

The multi-rope friction mining hoist consists of a wire rope placed on the leading wheel (the friction wheel), a lifting vessel suspended at both ends, and a balance hammer that can be suspended at one end. When the motor drives the leading wheel, the friction force on the transmission wire rope between the liner and the wire rope, which is installed on the leading wheel, can then lift and place heavy objects (Figure 1).

**Figure 2:**
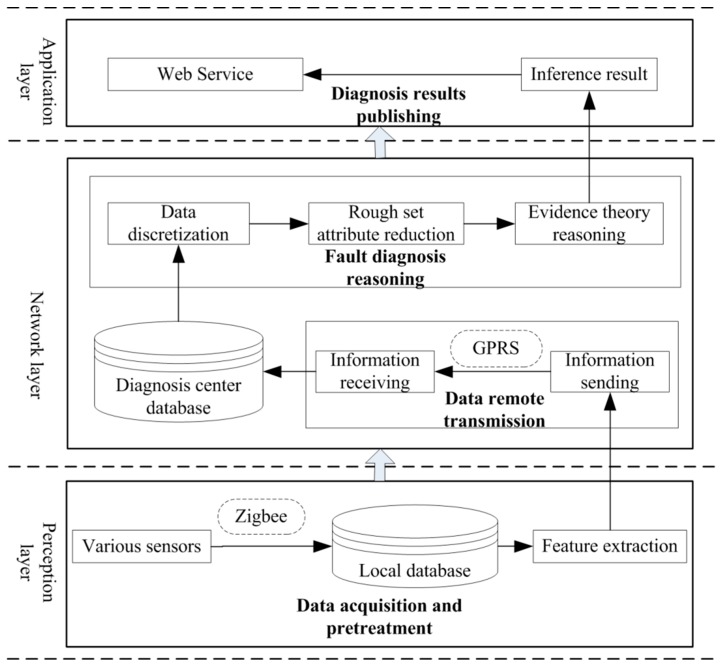
The system structure.

Based on the architecture of the IoT, in the present study we used the three-layer architecture known as the “sensor layer–network layer–application layer” and combined the working environment, system structure, and monitoring and diagnosis of the mine hoist. The IoT-based method for the fault diagnosis of mine hoist equipment was studied, and the corresponding system architecture is shown in Figure 2.

Also called the information acquisition layer, the perception layer is responsible for data input and knowledge acquisition. The data collected by the monitoring system, the structural parameter information of the hoist itself, existing diagnostic knowledge, and expert diagnostic knowledge are pre-processed into the next layer. The network layer includes two parts: information transmission and diagnostic reasoning.

The information transmission part mainly transferred information obtained by the perceptual layer, which includes a transmitter, receiver, and a wireless transmission module. To ensure that the application layer was more focused on the external release of the fault monitoring and diagnosis information, the network layer incorporated both the diagnosis and inference analysis of the fault data. A fault diagnosis method was used that has high efficiency, reasonable diagnosis, and high accuracy for reasoning and analyzing the transmitted information. Finally, the fault diagnosis results were obtained.

The application layer comprises the information release layer, which can be customized based on user needs, and the diagnosis results are released to the user interface.

### 2.1. Fault Monitoring System and Fault Diagnosis Knowledge Acquisition Model of Mine Hoist Layer

**Figure 3:**
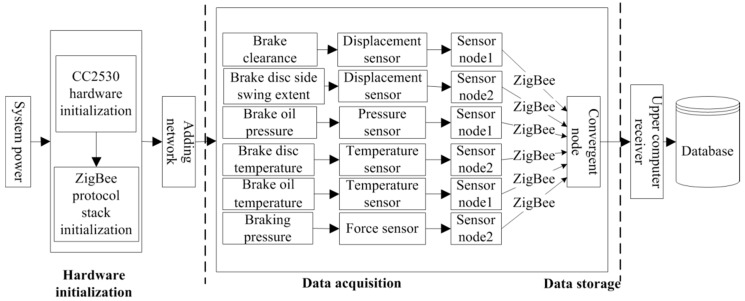
Internet of Things (IoT) perception layer structure.

The perception layer is responsible for data collection, wireless data transmission over short distances, reception, display, and data preservation. Based on ZigBee technology [21,22], we used SoC CC2530 as the core module of the sensor nodes and the converging nodes. Based on the data types and characteristics, the connections of different sensors and core modules were designed to obtain the corresponding sensor nodes. The ZigBee standard transmission protocol was selected to establish a star network topology. A line data acquisition system was employed to collect operational data [23,24,25]. Taking the hoist brake system as an example, the structure of the perception layer in the hoist fault diagnosis system based on the IoT is shown in Figure 3.

### 2.2. Network Information Transmission and Fault Diagnosis Model

**Figure 4:**
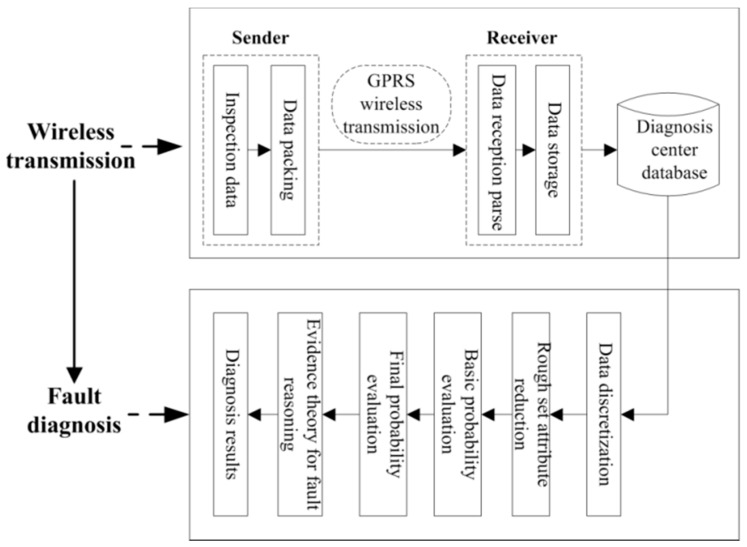
IoT network layer structure.

As a bridge to the IoT, the network layer provides technical support for long-distance remote monitoring, diagnosis, manipulation, and other data-supporting work. The network layer is mainly divided into two parts: one part manages long-distance wireless transmission, and the other performs data fault diagnosis. At the transmitter, data are transferred from the database wirelessly in the form of a package using a GPRS module over a mobile network. The receiving end parses and saves the data to the remote diagnostic center database. Based on rough set theory, a knowledge acquisition model of fault diagnosis rules was constructed for the hoister. First, the historical diagnosis dataset and domain experience knowledge were discretized. Then, the decision table was constructed, and the diagnosis rules were reduced. Thus, the resulting high-confidence diagnosis rules were stored in the knowledge library. Next, we used DSmT evidence theory to perform fault reasoning and to finally obtain the fault diagnosis result. The structure of the network layer is shown in Figure 4.

### 2.3. Release of Application Layer Information

The application layer mainly includes the fault release platform, which includes the fault release interface, fault type determination, fault cause analysis, solution proposal, and fault information preservation. As a direct display screen for man–machine interaction, the fault release interface clearly shows the types of and reasons for faults, in addition to providing reasonable solutions and storing the faults.

## 3. Key Fault Diagnosis Technologies

Neural networks are widely used for mechanical fault diagnosis [26]. By using data pre-processing combined with the Self-Organizing Maps(SOM) neural network and rough set theory, the basic probability assignment (BPA) of DSmT fault reasoning in the system can be effectively extracted, and the influence of subjective factors on this process can be reduced considerably. The aforementioned method outputs a more accurate BPA value, is objective, and improves fault reasoning in terms of objectivity and efficiency. By using the modified DSmT evidence theory to perform the uncertainty of fault reasoning, conflicting information can be retained, control factors in the reasoning process can be calculated objectively, the interference of subjective factors can be reduced, and uncertainties in fault diagnosis can be better resolved, causing the reasoning results to be more accurate. Moreover, this method can fully exploit the advantages of the three methods, ensure they complement each other, and combine the three methods to improve the efficiency and accuracy of the fault diagnosis. The combined fault diagnosis method involving the SOM neural network, rough set theory, and DSmT evidence was verified experimentally. Detailed descriptions of the key technologies used in the troubleshooting process are outlined below.

### 3.1. SOM Neural Network Discretization

When the rough set attributes are reduced, the object of reduction is usually discrete data, but the relevant information for a braking system is continuous. Data must be pre-processed by discretization to further reduce the data.

Many kinds of discrete methods have been introduced, such as the naive scaler algorithm, the fish swarm optimization clustering method, the self-organized feature mapping neural network (SOM neural network) method [27,28], the naive algorithm, the Boolean reasoning algorithm, the semi-naive algorithm, and the decision tree method. Different methods have their own unique advantages and drawbacks, and the validity of any discretization method cannot be applied to all data sets. In terms of discretization, the discretization effect of each discretization method will be very different. The disadvantage of many of these discretization methods is the subjective constraints. To overcome this drawback, autonomous learning should be considered first, and intelligent classification completed. The most commonly-used method is the SOM neural network method.

The most useful feature of SOM is that, after entering a data sample into the network, the network automatically calculates different intervals according to the previous parameter settings. Each interval has a different characteristic, and the parameters of the set have fixed principles that are not subject to the interference of the subjective consciousness [29]. For example, to determine the number of division intervals, mainly based on the status of the sample data to determine the type for the reduction of the attributes of the rough set, the fixed principle is the number of types of decision attributes.

**Figure 5:**
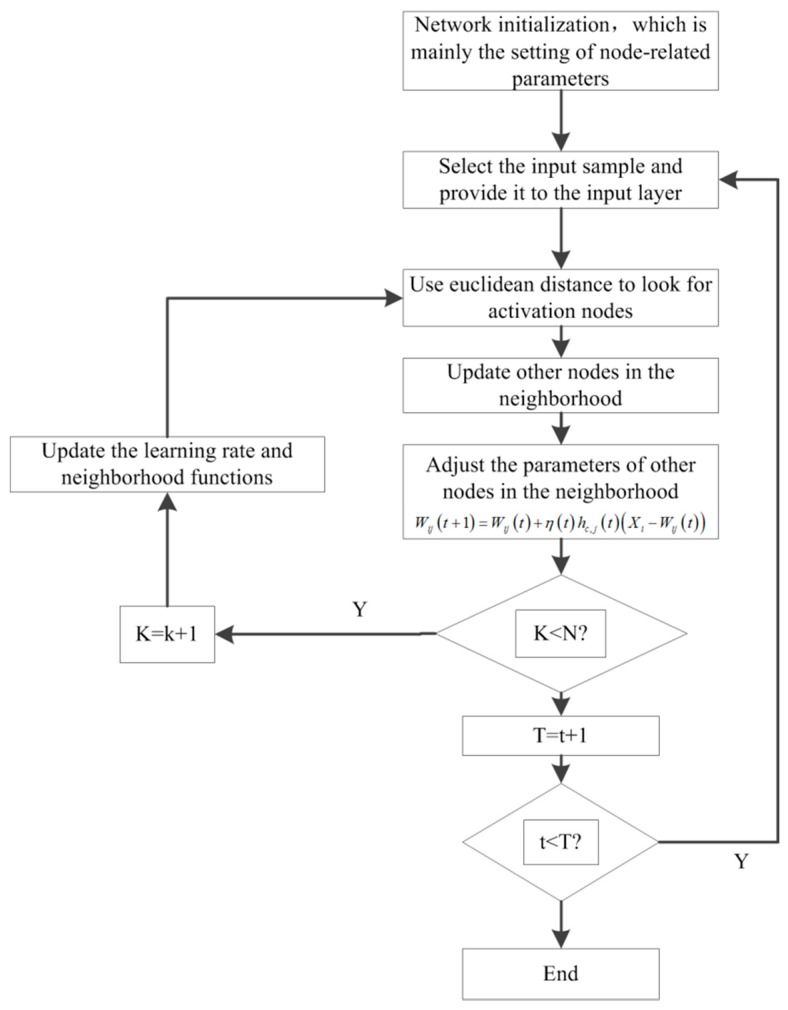
Flow chart of the SOM algorithm.

The process of implementing the SOM algorithm is shown in Figure 5.

When discretizing data using SOM, only the number of clusters must be specified because the other parameters are self-configured by the network. When the parameters related to the hoist and the condition attributes are discretized, the number of clusters is equal to the number of classes of decision attributes. This is an objective setting that reduces the interference of the subjective factors in addition to enhancing the effectiveness of discretization.

### 3.2. Rough Set Attribute Reduction Based on an Improved Difference Matrix

As the target object of the rough set is only the information data, the information data is not affected by other factors such as experience or expert knowledge. Therefore, the selected decision attribute can avoid subjectivity. This is advantageous from the viewpoint of data pre-treatment, uncertainty description, knowledge rule acquisition, and for the objective handling of data [30,31].

As a result of deep discussion regarding rough sets, different attribute reduction algorithms have been promoted, including data analysis, equivalence classification, the difference matrix method, and improved difference matrix method. The data analysis method and the equivalence classification method are rarely used in practical applications due to their disadvantages, which include high computational complexity, complex logic operations, and low reduction efficiency. The difference matrix method is not favorable for handling large or complex data, such as the calculation of conjunctive forms, and the resource wastage in this method leads to low reduction efficiency. The improved matrix in the differences method offers a good combination of the advantages of the above three methods, and mitigates their shortcomings. The differences method is a highly efficient and accurate attribute reduction method. After comprehensive consideration, we concluded that using the improved difference matrix method as the algorithm for reduction would help with the quick interpretation and calculation of the core number and reduce the sample data [32].

The improved difference matrix method is further processed based on the difference matrix: *C*(*S*) = (*c_ij_*):

(1) $$c_{ij}=\{\begin{array}{cc} \left(a\in A:a\left(x_{i}\right)\neq a\left(x_{j}\right)\right) & D\left(x_{i}\right)\neq D\left(x_{j}\right)\\ 0 & else \end{array},$$

where *i*, *j* = 1, 2, 3, …, *n*, which is also denoted as *c_ij_* for the difference element.

When using the improved difference matrix for reduction, the main goal is to first find the core attribute of a given attribute set. Each attribute set has core attributes. The difference matrix contains a single element attribute, and the kernel of this attribute is the set of these single elements. After finding the core of the attribute set, all differential elements containing the kernel elements in the discernibility matrix are reduced to 0. Then, the conjunctions of each column are found, merged, and reduced to obtain the final reduction results.

Compared with the improved matrix, the biggest feature is that the improved difference matrix plays a core role. Firstly, the core attributes are found, and then various elements containing the core attributes are changed to 0. After simplification, most of the different elements in the difference matrix become 0, which greatly simplifies the conjunctive form of each column of difference elements. Hence, the reduction of the conjunctive form becomes simpler and more convenient. Moreover, the method can find the core attribute of the attribute set, which is useful for subsequent data processing.

### 3.3. Improved Dempster-Shafer (D-S) Evidence Theory

For ordinary reasoning decision-making problems, evidential theory offers a clear reasoning structure, high decision diagnosis accuracy, convenient operation, and wide scope of application. However, the theory has some shortcomings, of which the most prominent is that the evidence must be independent.

When the conflict rate between the evidence is not high, the synthesis rules of the D-S evidence theory can be used effectively. However, when a certain conflict exists between the evidence, the synthetic rules directly remove the conflicting information. Therefore, the information [33] cannot be considered in an all-round way. The combination characteristic of DSmT can solve this problem effectively. The conflict rules are omitted, and the loss of effective information can be avoided [34,35].

The main difference between DSmT and Dempster-Shafer Theory(DST) is as follows: Hypothesis Θ = {θ1, θ2}, then for DST: m(θ1) + m(θ2) + m(θ1 ∪ θ2) = 1, and for DSmT: m(θ1) + m(θ2) + m(θ1 ∪ θ2) + m(θ1 ∩ θ2) = 1.

The modified DSmT combination rule is as follows:

(2) $$m_{12}\left(X\right)=\frac{\sum_{\begin{array}{l} A,B\in D^{\Theta },\\ A\cap B=X \end{array}}m_{1}\left(A\right)m_{2}\left(B\right)+P\left(X\right)}{1-k^{\prime }},\forall X\neq \phi \in D^{\Theta },$$

where *X* is an empty set, *m*_12_(*X*) = 0. Among them:

(3) $$\sum_{X\in D^{\Theta }}P\left(X\right)=\left(1-\sigma \right)k,$$

(4) $$k=\sum_{A,B\in D^{\Theta },A\cap B=\varphi }m_{1}\left(A\right)m_{2}\left(B\right),$$

where *σ* is the control factor, and *K*’ = *σK*, where *K* reflects the size of the conflict. *σ* = Sim(*m*_1_, *m*_2_), Sim(*m*_1_, *m*_2_) = 1 − Dis(*m*_1_, *m*_2_), where Dis(*m*_1_, *m*_2_) is the Jousselme distance between the evidence:

(5) $$\mathrm{Dis}\left(m_{1},m_{2}\right)=\sqrt{\frac{1}{2}{\left(m_{1}-m_{2}\right)}^{T}D\left(m_{1}-m_{2}\right)},$$

(6) $$D\left(A,B\right)=\frac{\left|A\cap B\right|}{\left|A\cup B\right|},\forall A,B\in D^{\Theta },$$

Through further modification of the DSmT combination rules, the uncertainty problem can be reasoned more objectively. Regardless of whether the information contains conflicting content, the improved rules treat each piece of information objectively and do not ignore any information. The entire reasoning process is devoid of human factor interference, so the result is objective and reliable.

## 4. Experimental Verification

**Figure 6:**
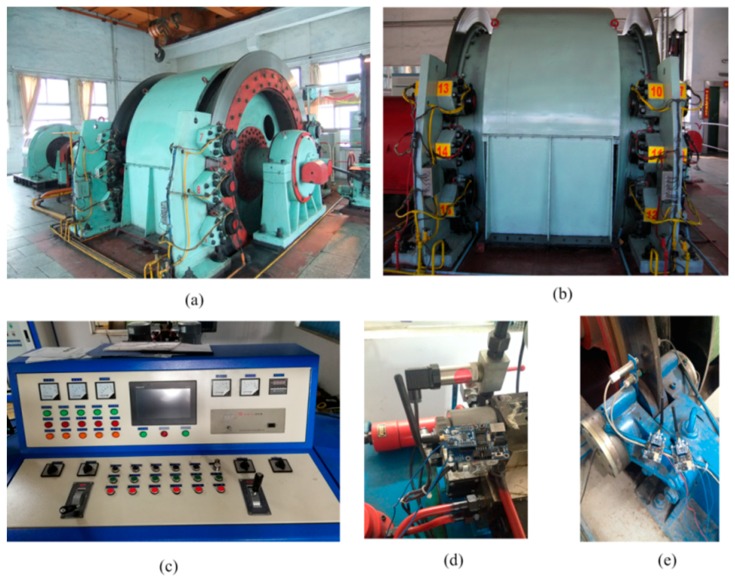
Field diagram of test equipment. (**a**) Main shaft device of a hoist; (**b**) brake device; (**c**) operating table; (**d**) wireless acquisition of the pressure signal in a hydraulic station; and (**e**) wireless acquisition of brake disc space.

Taking a 2JTP-1.2 double drum single rope friction hoist as an example, the experimental research was performed, as shown in Figure 6.

### 4.1. Perception Layer Test

**Figure 7:**
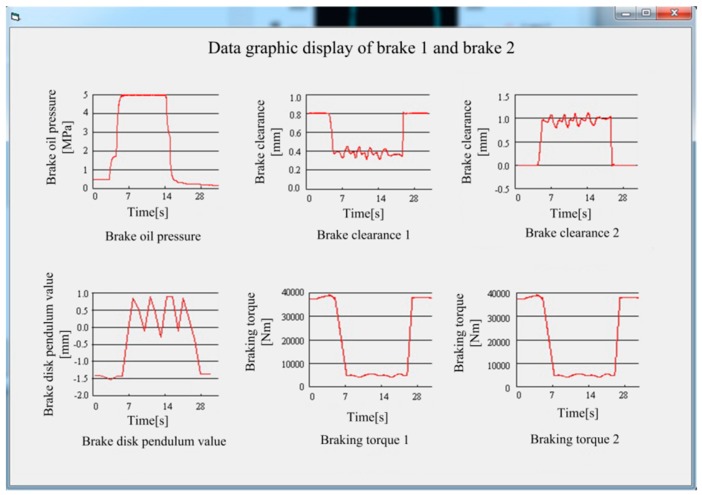
Detailed data of brakes 1 and 2.

The braking system was used as the experimental research object. The hoist uses four pairs of eight-disc brakes for braking. By installing the sensors above brakes 1 and 2, we obtained 15 data points per second, and the obtained results are shown graphically on the interface in Figure 7.

**Figure 8:**
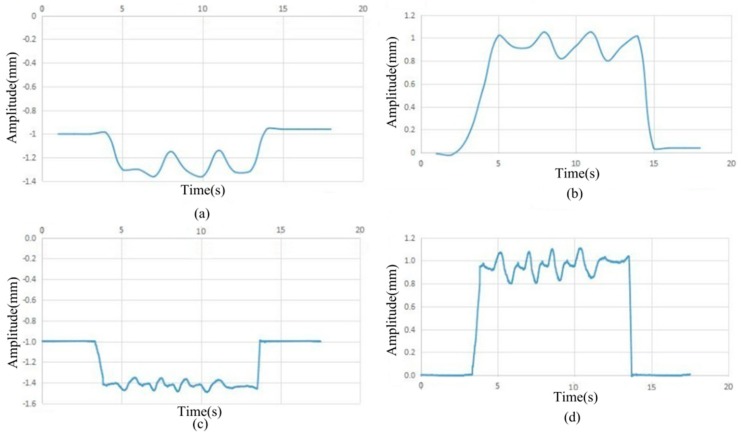
Comparison of experimental data. (**a**,**c**) time domain variation in the collected gap data display of the data collected using KingView; (**b**,**d**) time domain variation in the collected gap data display of the data collected using the system.

To check the accuracy of the collected signals, the data were collected synchronously using the traditional method involving collecting cards and the Kingview display. The time domain variation in the collected gap data is shown in Figure 8. Graphs (a) and (c) denote the display of the data collected using KingView. Graphs (b) and (d) denote the display of this system.

From the specific changes in Figure 8 in (a) and (b), and (c) and (d), the general changes in the patterns in (b), (d), (a), and (c) are consistent, indicating that the collected data were roughly the same. The amplitude of the graphics is visible, and especially in (b) and (d), the amplitude of the graphics are clearer. The data acquisition is more accurate and more valuable for the important parameter of brake shoe clearance. In sum, the results of this system are not only consistent with those obtained using the traditional method, but they are also more accurate, clear, and practical.

### 4.2. Network Layer Test

To simulate the faults of the braking system, the following four groups of experiments were conducted. (1) Excessive residual pressure: The residual pressure of two pairs of brakes was increased to 0.8 MPa; (2) Excessive brake shoe clearance: The brake shoe clearance of a pair of brakes was increased to increase the gap between brake shoes 3 and 4; (3) Hybrid fault: The gap between brake shoes 3 and 4 was increased, and the residual pressure of the two pairs of brakes was increased by 0.7 MPa; (4) Normal: Data were collected under normal conditions.

A data acquisition system was used to collect data on the brake shoe gap, disc spring force (computable brake torque), and speed under the normal condition and the failure states listed above for (1)–(3).

According to the rough set reduction idea, four fault types were defined, that is, the decision attribute was defined. The four decision-making attributes were as follows: T1—gap between brake tiles (3, 4), T2—excessive residual pressure (0.8 Mpa); T3—mixed failure (residual pressure + gap); and T4—normal. Considering the reasonable selection of the condition attributes according to the test conditions, nine conditions were defined as follows: C1—brake shoe clearance 1; C2—brake shoe clearance 2; C3—brake shoe clearance 3; C4—brake shoe clearance 4; C5—braking torque 1; C6—braking torque 2; C7—braking torque 3; C8—braking torque 4; and C9—speed.

After defining the decision attributes and the conditional attributes, we selected the sample data and the data to be measured. From the four groups of tests, each of the corresponding four decision attributes was selected from each of the two datasets to form a sample matrix, and a set of data was selected from each type of fault data as the data to be detected. The sample data are provided in Table 1.

In Table 1, the first eight sets of data were used as the sample data, and the last four sets of data were used as the fault data to be measured. The discretization results were obtained after the data were discretized using the SOM neural network and the redundant data were removed. According to the analysis in Section 4.1, we directly imported the sample data into the MATLAB software and set the number of clusters to four. Thus, four was the number of decision attributes. The discretization results are listed in Table 2.

Difference matrix analysis was performed on the first eight sets of fault data outlined in Table 2.

$$\left\{\begin{array}{ccccccc} 0 & 0 & 0 & & & & \\ 134579 & 0 & 0 & & & & \\ 14 & 134579 & 0 & & & & \\ \cdots & \cdots & 134579 & 0 & & & \\ \vdots & \vdots & \vdots & \vdots & & 0 & \\ 134579 & 7 & 134579 & 57 & \cdots & 134579 & 0 \end{array}\right\}$$

From the difference matrix, the single elements were 1, 5, and 7, so the core of the sample data was {C1, C5, C7}, and the terms of the elements containing the core in the difference matrix were reduced to 0. Only 34 remained. Therefore, the final reduction result was RED1 = {C1, C3, C5, C7} and RED2 = {C1, C4, C5, C7}. The reduction results are listed in Table 3.

The attribute importance of RED1 = {C1, C3, C5, C7} relative to the decision attribute D was calculated separately to obtain SGF (C1, D) = 1 − 5/8 =3/8, SGF (C3, D) = 1 − 6/8 = 2/8, SGF (C5, D) = 1 − 6/8 = 2/8, and SGF (C7, D) = 1 − 4/8 = 4/8. The normalized processing could be assigned weights, respectively, of 0.273, 0.182, 0.182, and 0.364.

The attribute importance of RED2 = {C1, C4, C5, C7} relative to the decision attribute D was calculated separately, obtaining SGF1 (C1, D) = 1 − 4/8 = 4/8, SGF1 (C4, D) =1 − 6/8 = 2/8, SGF1 (C5, D) =1 − 6/8 = 2/8, and SGF1 (C7, D) = 1 − 4/8 = 4/8. The normalized processing assigned weights were 0.333, 0.167, 0.167, and 0.333, respectively.

C1, C3, C5, and C7 evidence r1, r2, r3, r4 and C1, C4, C5, C7 evidence R1, R2, R3, R4, respectively. Synthetic evidence *r* and *R* and four kinds of phenomena were identified as frame elements Θ1, Θ2, Θ3, and Θ4. Under the RED1 and RED2 conditions, the basic probability assignment for group 9 data is summarized in Table 4.

Considering the different degrees of importance of D and the weight value to obtain the final evidence *r*, *R*, the synthesis results obtained are shown in Table 5.

The *r* and *R* evidence fusion results were used as two sets of values for the basic confidence function, and they were subsequently converged using the modified DSmT evidence theory. The fusion results are listed in Table 6.

As shown in Table 6, the result of T1 was the largest, so the fault diagnosis data with excessively large gaps could be reasoned out. This was consistent with the actual fault conditions in the brake shoe gap, which means that the diagnostic results were accurate. The same algorithm was used to judge the data of groups 10, 11, and 12, and the results of the evidence fusion are shown in Table 7, Table 8 and Table 9, respectively.

These tables show that the fusion results of T2, T3, and T4 were the greatest. The fault types were judged to be T2, T3, and T4, which correspond to the mixed failure of the residual pressure, clearance of the brake shoe and the residual pressure and normal state, respectively. The fault reasoning results conform with the actual data collected, which means the accuracy of the diagnosis was high.

### 4.3. Application Layer Test

**Figure 9:**
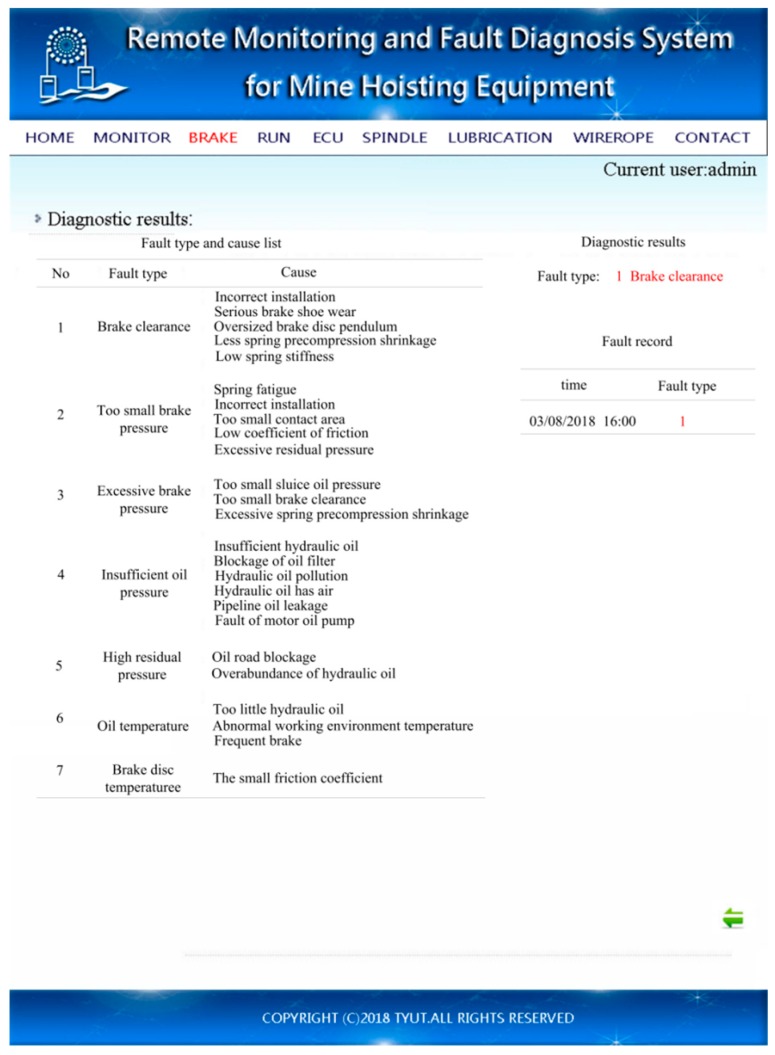
Interface of fault release.

The application layer was mainly used for fault release. Taking the “gap is too large” fault as an example, the application layer fault release is shown in Figure 9. The diagnosis results show 1, and the diagnosis result is fault 1. Compared to the type of fault, the brake shoe gap fault and the corresponding causes of fault and maintenance suggestions can be found one at a time.

A comprehensive analysis of the cause of failure shows five possible reasons for the failure of the brake shoe clearance. The five reasons were analyzed individually. As the physical quantities of the brake disc were measured directly, the deflection value of the brake disc was within the specified range according to the measurement results, which means this cause was eliminated. The spring force monitoring data showed that the spring force was significantly lower than that in the normal state, indicating that a reduction of the spring pre-pressure shrinkage or decrease in spring stiffness may have been the cause of the failure. The braking effect was obviously improved, and the braking force was restored to normal after increasing the pre-pressure shrinkage value in the test, which means a judgment was made. The brake shoe clearance was too large, possibly due to the spring preload shrinkage. If normal installation was completed, improper installation of the hoist could be used only as a reference when there is no damage to the fixed facilities of the various parts of the hoist. As for the serious wear and tear of the brake shoe, the clearance between the two brake shoes obviously increased, and when the brake shoe was checked, there was no obvious wear. Hence, the gap between two brake shoes was too large to be caused by abrasion. Based on the above analysis, the main cause of the brake shoe gap fault was the reduction in spring preloading shrinkage, which was consistent with the actual operation of the brake shoe clearance fault simulation.

## 5. Conclusions

Based on the network structure of the IoT, a fault diagnosis framework for mine hoisting equipment based on intelligent combination was constructed. Based on ZigBee short-range wireless communication technology, a collaborative information acquisition system targeting key components of mine-lifting equipment was established, and real-time data acquisition was realized. A transmission and reception platform based on GPRS long-distance wireless data transmission was established to perform real-time data transmission. Based on multi-disciplinary crossover and fusion, the traditional fault diagnosis method for the mine lifting braking system was modified using a variety of fault diagnosis algorithms. A fault reasoning framework based on various algorithms was established, and this framework was found to be highly efficient, accurate, and able to perform intelligent fault diagnosis of mine hoisting equipment.

## Figures and Tables

**Table 1:** Experimental data.

Attribute Sample	Condition Attribute									Decision Attribute
	C_1_	C_2_	C_3_	C_4_	C_5_	C_6_	C_7_	C_8_	C_9_	
1	0.91	1.19	2.27	2.24	−428.95	259.38	158.62	2.56	1	T_1_
2	0.00	0.01	−0.05	0.00	33344.94	37230.60	20189.96	20964.40	0	T_1_
3	1.12	1.82	2.26	1.61	512.33	−504.01	−102.75	0.92	1	T_2_
4	0.09	−0.10	0.06	0.01	24393.81	24159.86	22722.8	21360.25	0	T_2_
5	0.62	0.69	2.23	2.81	−1151.1	−67.1	−90.9	−30.48	1	T_3_
6	0.00	0.03	0.04	−0.02	25899.6	28704.5	13978.93	13391.46	0	T_3_
7	0.90	1.20	1.39	1.23	1867.37	−1090.4	1919.43	1579.2	1	T_4_
8	−0.08	−0.18	−0.14	0.11	40283.35	37423.8	38054.61	35896.8	0	T_4_
9	0.92	1.13	2.34	2.28	422.97	−159.50	325.85	−96.40	1	T_1_
10	0.09	−0.11	0.05	0.01	24434.07	24010.88	22024.2	21571.25	0	T_2_
11	0.03	0.04	−0.02	−0.05	23962.5	26594.26	13667.04	12984.78	0	T_3_
12	0.88	1.23	1.34	1.52	−185.84	−1565.8	−85.86	354	1	T_4_

**Table 2:** Data discretization.

Attribute Sample	Condition Attribute						Decision Attribute
	C_1_	C_3_	C_4_	C_5_	C_7_	C_9_	
1	3	4	4	1	1	4	T_1_
2	1	1	1	4	3	1	T_1_
3	4	4	3	1	1	4	T_2_
4	1	1	1	3	3	1	T_2_
5	2	4	4	1	1	4	T_3_
6	1	1	1	3	2	1	T_3_
7	3	3	3	1	1	4	T_4_
8	1	1	1	4	4	1	T_4_
9	3	4	4	1	1	4	T_1_
10	1	1	1	3	3	1	T_2_
11	1	1	1	3	2	1	T_3_
12	3	3	3	1	1	4	T_4_

**Table 3:** Reduction results.

Attribute Sample	RED_1_				RED_2_				D
	C_1_	C_3_	C_5_	C_7_	C_1_	C_4_	C_5_	C_7_	
1	3	4	1	1	3	4	1	1	T_1_
2	1	1	4	3	1	1	4	3	T_1_
3	4	4	1	1	4	3	1	1	T_2_
4	1	1	3	3	1	1	3	3	T_2_
5	2	4	1	1	2	4	1	1	T_3_
6	1	1	3	2	1	1	3	2	T_3_
7	3	3	1	1	3	3	1	1	T_4_
8	1	1	4	4	1	1	4	4	T_4_
9	3	4	1	1	3	4	1	1	T_1_
10	1	1	3	3	1	1	3	3	T_2_
11	1	1	3	2	1	1	3	2	T_3_
12	3	3	1	1	3	3	1	1	T_4_

**Table 4:** Basic Probability Assignment (BPA).

Element Evidence	Θ_1_	Θ_2_	Θ_3_	Θ_4_
r_1_	1/2	0	0	1/2
r_2_	1/3	1/3	1/3	0
r_3_	1/4	1/4	1/4	1/4
r_4_	1/4	1/4	1/4	1/4
R_1_	1/2	0	0	1/2
R_2_	1/2	0	1/2	0
R_3_	1/4	1/4	1/4	1/4
R_4_	1/4	1/4	1/4	1/4

**Table 5:** Evidence fusion results.

Element Evidence	Θ_1_	Θ_2_	Θ_3_	Θ_4_
*r*	0.334	0.197	0.197	0.273
*R*	0.375	0.125	0.209	0.292

**Table 6:** First fusion result.

Element Evidence	Θ_1_ (T_1_)	Θ_2_ (T_2_)	Θ_3_ (T_4_)(T_1_T_2_)	Θ_4_ (T_5_)
*r* + *R*	0.467	0.092	0.154	0.297

**Table 7:** Second fusion result.

Element Evidence	Θ_1_ (T_1_)	Θ_2_ (T_2_)	Θ_3_ (T_3_)(T_1_T_2_)	Θ_4_ (T_4_)
*r* + *R*	0.300	0.502	0.148	0.049

**Table 8:** Third fusion result.

Element Evidence	Θ_1_ (T_1_)	Θ_2_ (T_2_)	Θ_3_ (T_3_)(T_1_T_2_)	Θ_4_ (T_4_)
*r* + *R*	0.038	0.113	0.816	0.038

**Table 9:** Fourth fusion result.

Element Evidence	Θ_1_ (T_1_)	Θ_2_ (T_2_)	Θ_3_ (T_3_)(T_1_T_2_)	Θ_4_ (T_4_)
*r* + *R*	0.269	0.096	0.057	0.576
